# Supplementary material for: Development of a Bispecific Nanobody Targeting CD20 on B-Cell Lymphoma Cells and CD3 on T Cells
Source: Vaccines (Basel). 2022 Aug 17;10(8):1335. doi: 10.3390/vaccines10081335 (PMC9413575; doi:10.3390/vaccines10081335)
Supplement: Supplementary file 1 [file vaccines-10-01335-s001.zip › vaccines-1835063-supplementary.pdf]

Supplementary Material

Supplementary Figures

Figure S1

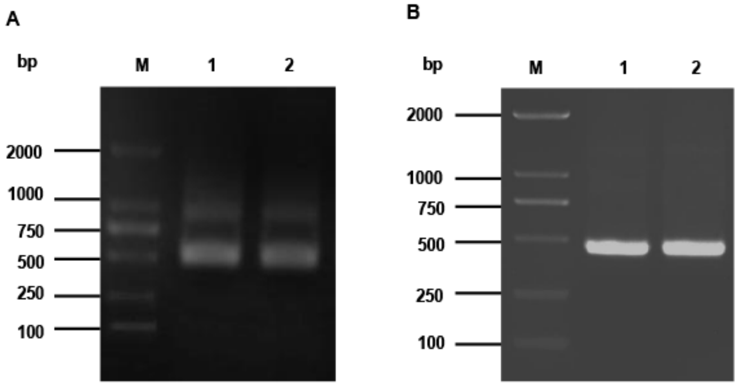

Figure S1. PCR amplification of VHH gene fragment. The first round of PCR amplified with cDNA as template to obtain the gene sequence of the leading signal sequence to the CH2 region, and which contains the 900 bp (VH-CH1-CH2) and 600 bp (VHH-CH2) fragments **(Figure S1A)**. The second round of PCR was performed to obtain the full-length of VHH gene from FR1 to FR4 with fragment size of approximately 400 bp **(Figure S1B)**.

Figure S2

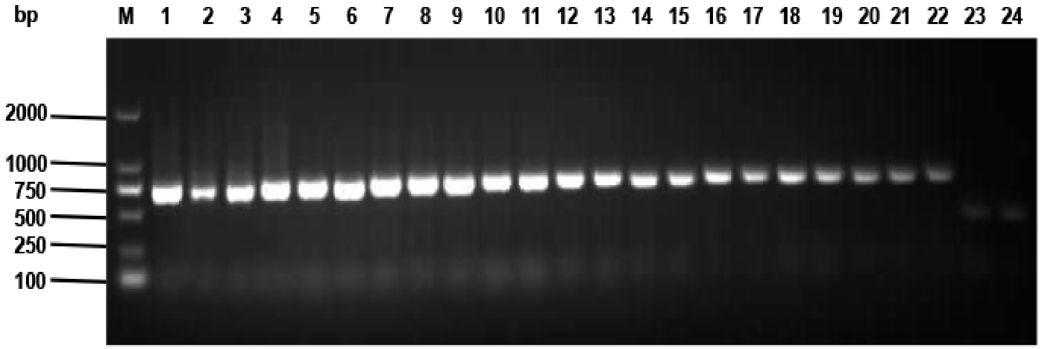

Figure S2. Identification of phage display antibody library. The antibody library prepared was diluted from 10^1 to 10^{10}, and coated in 2×YT-AG solid medium, cultured overnight at 37°C, and counted the colonies in the next day. The calculated size of phage antibody library was 1.2×10^8. Twenty-four clones were randomly selected from 2×YT-AG solid medium, and 22 clones among them could obtain the target band of approximately 600 bp by PCR amplification, and the transformation efficiency of antibody library was 91.7% as calculated.

Figure S3

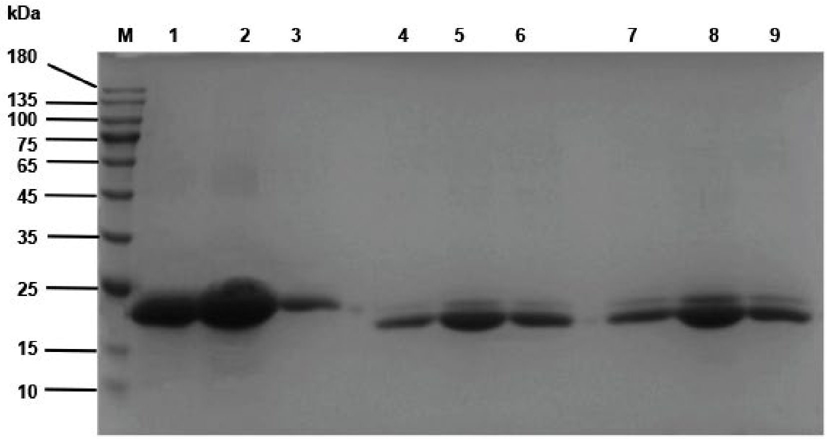

Figure S3. Expression and purification of recombinant sdAb. The bacterial lysate containing expressed sdAb was prepared by ultrasonication. The protein affinity purification of the bacterial lysate was performed with Ni-NTA Sefinose™ Resin. SDS-PAGE analysis showed that a concentrated band with a size of approximately 20 kDa was appeared, which was consistent with the expected molecular weight, and indicating that the recombinant sdAb with high purity was obtained. Lane M, Protein Marker (10-180 kDa). Lane 1-3,4-6,7-9, purified sdAbs.

Figure S4

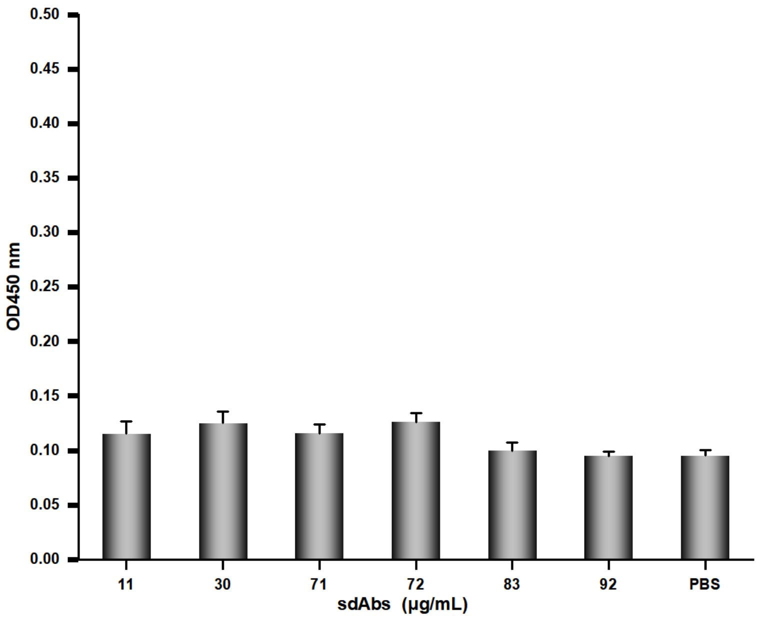

Figure S4. The binding activity of recombinant sdAbs to unrelated oligopeptides was measured by indirect ELISA. Recombinant sdAbs 11, 30, 71, 72, 83 and 92 were added to microtiter plates precoated with human β-amyloid (Aβ_{1-42}) oligopeptide at a final concentration of 5 μg/mL, and binding of recombinant sdAb was detected with an HRP-conjugated 6×His-tagged mouse monoclonal antibody. PBS was used as a blank control.

Figure S5

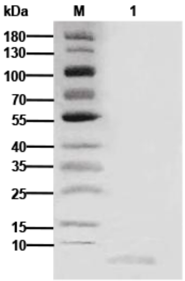

Figure S5. The specificity of the recombinant sdAb was detected by Western blotting. The human CD20 extracellular polypeptide was separated by SDS–PAGE electrophoresis and transferred to PVDF membranes, and the recombinant sdAb was used as the primary antibody, while the HRP-conjugated 6×His-tag mouse monoclonal antibody was used as the secondary antibody. There was a target band at approximately 6 kDa, which was consistent with the expected molecular weight, indicating that the recombinant sdAb could specifically bind to the CD20 extracellular polypeptide. Lane M, Protein Marker (10-180 kDa). Lane 1, CD20 polypeptide.

Figure S6

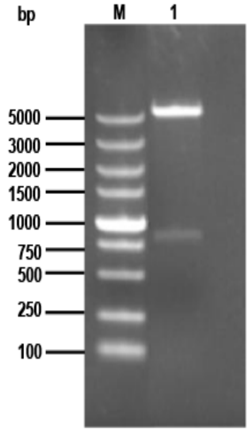

Figure S6. Identification of recombinant anti-CD20/CD3 BsNb plasmid with enzyme digestion. The synthesized CD20 sdAb-linker-CD3 sdAb gene sequence was ligated to pET-22b (+) vector, and transformed into E. coli BL21 (DE3) competent cells. The plasmid was extracted, and the target band approximately 830 bp and vector band approximately 5,400 bp were identified by *Not* I and *Nco* I double enzyme digestion.
